# Genetic and QTL mapping in African bermudagrass

**DOI:** 10.1002/tpg2.20073

**Published:** 2021-03-03

**Authors:** Shuhao Yu, Tilin Fang, Hongxu Dong, Liuling Yan, Dennis L. Martin, Justin Q. Moss, Charles H. Fontanier, Yanqi Wu

**Affiliations:** ^1^ Dept. of Plant and Soil Sciences Oklahoma State Univ. 371 Agriculture Hall Stillwater Oklahoma 74078 USA; ^2^ Dept. of Plant and Soil Sciences Mississippi State Univ. 358 Dorman Hall Starkville Mississippi 39762 USA; ^3^ Dept. of Horticulture and Landscape Architecture Oklahoma State Univ. 358 Agriculture Hall Stillwater Oklahoma 74078 USA

## Abstract

*Cynodon transvaalensis* Burtt‐Davy is frequently used to cross with *C. dactylon* Pers. in the creation of F_1_ hybrid cultivars that are some of the most widely used in the worldwide turf industry. However, molecular resource development in this species is limited. Accordingly, the objectives of this study were to construct a high‐density genetic map, and to identify genomic regions associated with establishment rate. In this study, we constructed the first high‐density linkage map for African bermudagrass using a genotyping by sequencing approach based on 109 S_1_ progenies. A total of 1,246 single nucleotide polymorphisms and 32 simple sequence repeat markers were integrated in the linkage map. The total length of nine linkage groups was 882.3 cM, with an average distance of 0.69 cM per interval. Four genomic regions were identified to be associated with sod establishment rate. The results provide important genetic resources towards understanding the genome as well as marker‐assisted selection for improving the establishment rate in bermudagrass breeding.

## Core Ideas

- Developed the first African bermudagrass high‐density linkage map.
- Identified four genomic regions associated with turf establishment rate in the species.

**Abbreviations**
ER: establishment rate
GBS: genotyping‐by‐sequencing
IM: interval mapping
LG: linkage group
LOD: logarithm of the odds
MQM: multiple QTL model
NGS: next‐generation sequencing
PCR: polymerase chain reaction
PP: primer pair
QTL: quantitative trait loci
SDAF: single‐dose amplified fragment
SDRF: single‐dose restriction fragment
SNP: single nucleotide polymorphism
SSR: simple sequence repeat

## 1 INTRODUCTION

African bermudagrass (*Cynodon transvaalensis* Burtt‐Davy) is originated in southwestern Transvaal and the northern part of the central Cape Province of South Africa (Harlan, de Wet, & Rawal, 1970a). The species has distinct morphological features, including unique yellowish‐green color, slender stolons, short internodes, fine‐textured leaves, and small stature as compared with other species in *Cynodon* L.C. Rich. (Harlan, de Wet, Huffine, & Deakin, 1970b). A small number of African bermudagrass cultivars such as Florida and Uganda were used on putting greens and tennis courts (Juska and Hanson, 1964). The usage of African bermudagrass as turfgrass is limited due to the high demand for water and fertilizers, reduced turf quality in summer, and purple color under cool temperatures (Taliaferro, 1992). However, the primary use of African bermudagrass is to serve as a parent to cross with tetraploid or hexaploid common bermudagrass to create interspecific F_1_ hybrid cultivars (Burton, 1991; Taliaferro et al., 2006). Numerous hybrid cultivars have been widely used in the turf industry across the world since the 1960s. African bermudagrass contributes genes for several important traits related to turfgrass performance, including fine leaf blades, high sod density, tolerance to low mowing heights, and cold hardiness (Harlan et al., 1970a).

The base chromosome number of *Cynodon* species is nine (Forbes & Burton, 1963) and an estimated genome size of *C. transvaalensis* (2*n *= 18) is ∼540 Mbp in the haploid genome (Taliaferro & Lamle, 1997). The species is a largely self‐incompatible, cross‐pollinated taxon (Burton & Hart, 1967; Richardson, Taliaferro, & Abring, 1978). Kenworthy et al. (2006) estimated the broad‐sense heritability of 21 African bermudagrasses traits related to turf performance, inflorescence, and morphology as having a range from 0.42 to 0.96, indicating certain traits can be improved through recurrent selection. Studies have been conducted to quantify African bermudagrass genetic diversity with DNA markers (Wu, Taliaferro, Bai, & Anderson, 2005; Zhang et al., 1999). However, African bermudagrass molecular resource development, such as the construction of linkage maps, lags behind some other turfgrass species, like zoysiagrass (*Zoysia* spp.) (Huang et al., 2016; Wang et al., 2015) and St. Augustinegrass [*Stenotaphrum secundatum* (Walt.) Kuntze] (Yu, Kimball, & Milla‐Lewis, 2018). Linkage maps are one of the most important molecular tools in genetic and genomic research. To date, three African bermudagrass genetic linkage maps have been published based on one population (Bethel et al., 2006; Harris‐Shultz, Schwartz, Hanna, & Brady, 2010; Khanal et al., 2017). The most recent map was composed of 125 single‐dose restriction fragments (SDRF) and simple sequence repeat (SSR) markers (Khanal et al., 2017) and no high‐resolution linkage map is available for African bermudagrass. Therefore, in order to advance the efficiency for mapping and discovery of quantitative trait loci (QTLs)/genes in African bermudagrass, a high‐resolution linkage map is required (Mammadov, Aggarwal, Buyyarapu, & Kumpatla, 2012).

Single nucleotide polymorphisms (SNPs) are the most abundant DNA polymorphisms that can be utilized as molecular markers to construct linkage maps (Mammadov et al., 2012). Next‐generation sequencing (NGS) technologies provide efficient methods for discovering SNP markers in a fast and affordable way for both model and non‐model species (Wang et al., 2015). Genotyping‐by‐sequencing (GBS), an approach based on NGS, has been developed to generate a large volume of SNPs using appropriate methylation‐sensitive restriction enzymes to digest genomic DNA, while avoiding repetitive genomic regions to reduce the complexity of targeted genomes (Elshire et al., 2011). The GBS approach has been applied in SNP development and linkage map construction in many grass species used for turf, biomass, and forage, such as switchgrass (*Panicum virgatum* L.) (Fiedler et al., 2015), zoysiagrass (Holloway et al., 2018), St. Augustinegrass (Yu et al., 2018), and Chinese silvergrass (*Miscanthus sinensis* Andersson) (Dong et al., 2018; Liu et al., 2016). However, to date, no African bermudagrass linkage map has been constructed with SNP markers.

As popular interspecific hybrid bermudagrass (*C. dactylon*  ×  *C. transvaalensis*) cultivars such as Tifway, Latitude 36, Northbridge, Tahoma 31 and TifTuf are sterile, they can only be vegetatively propagated (Schwartz et al., 2018; Wu et al., 2013; Wu et al., 2014; Wu et al., 2019). Therefore, a fast establishment rate is critical to sod and sprig production. However, information on the genetic basis for controlling establishment rate is not available. Accordingly, the objectives of this study were to construct a high‐density genetic linkage map, and to identify QTLs associated with establishment rate.

**Core ideas**

- Developed the first African bermudagrass high‐density linkage map.
- Identified four genomic regions associated with turf establishment rate in the species.

## 2 MATERIALS AND METHODS

### 2.1 Plant materials

A mapping population was created by selfing a *C. transvaalensis* genotype OKC1163 (NTEP, 2017). The parent plant was planted into a 25 cm by 50 cm tray with greenhouse soil (Berger BM2, Berger, QC, Canada) in a greenhouse on Agronomy Farm, Oklahoma State University (OSU) in the spring of 2014. The temperature regime was maximum 35/21 °C (day/night). Sixteen hours of photoperiod to meet the minimal 25 mol m^−2^ d^−1^ daily light integral. The plant was placed on a bench that was relatively isolated from other bermudagrass plants, allowing it to produce selfed seed. Mature inflorescences were hand collected from the parent plant in June 2014 and stored after drying at ambient temperature for two weeks. Germination of 600 seeds was carried out in the greenhouse and seedlings were transplanted into 3.8 cm diameter cone‐tainers in March 2017. Initial visual screening to exclude presumed interspecific crossed progeny was conducted two months after germination based on typical *C. transvaalensis* morphological characteristics (i.e., fine leaf blades, yellowish‐green color, and short internodes). One‐hundred twenty‐four plants were selected from more than 300 putative selfed progeny.

Accurate selfed progeny identification was performed using SSR markers to remove unintended crossed progeny. Approximately 1 g of fresh leaf tissue from each of the selected progeny plants in the greenhouse was collected and immediately placed in an icebox and then stored in a −80 °C freezer. A tissue homogenizer (Geno/Grinder; SPEX SamplePrep, Metuchen, NJ) was used to grind leaf samples for 90 seconds at 1,500 RPM. DNA was extracted following a modified phenol‐chloroform extraction method (Nalini, Jawali, & Bhagwat, 2004). A NanoDrop ND‐1000 spectrophotometer (Thermo Fisher Scientific, Waltham, MA) was used to quantify DNA concentration and each DNA sample was then diluted to 10 ng μl^−1^ in preparation for polymerase chain reaction (PCR). Eleven *C. transvaalensis* SSR primer pairs (PPs) (CTGA7‐1983/1984, CTGA7‐1987/1988, CTGA7‐1993/1994, CTGA7‐1995/1996, CTAAG8‐2499/2500, CTAAG8‐2501/2502, CTAAG8‐2503/2504, CTAAG8‐2505/2506, CTAAG8‐2507/2508, CTAAT1‐2509/2510, CTAAT1‐2513/2514) developed by Tan et al. (2012) were employed to genotype the progeny. The PCR and gel electrophoresis were conducted following the procedures described by Fang et al. (2015). Progeny with different bands from the parent were identified as cross‐pollinated, which were excluded from the study. In total, 109 self‐pollinated progenies were retained for the following experiments.

### 2.2 Experimental design, establishment, and management in a replicated field trial

The 109 first‐generation selfed (S_1_) progenies and the parent were grown in 3.8 cm diameter cone‐tainers with daily irrigation and bi‐weekly fertilization with 12–4–8 (N–P–K) (Miracle Gro Lawn Products Inc., Marysville, OH) in the greenhouse since March 2017. To collect phenotypic data for turf establishment rate, greenhouse grown plants were transplanted to a nursery on the OSU agronomy farm (36°12' N lat.; 97°08' W long.), Stillwater, OK on 20 June 2017. The experiment was arranged as a randomized complete block design with three replications. Plot size was 0.9 m by 0.9 m with 0.3 m wide alleys between neighboring plots. Each plot was established with one plant in the center. The soil type was a Kirkland silt loam (fine, mixed, superactive, thermic Udertic Paleustoll). Ronstar 2G herbicide (oxidiazon; Bayer Environmental Science, Montvale, NJ) was applied at 2.2 kg ha^−1^ of active ingredient one day after transplanting to prevent weeds. Based on the results of a soil chemical analysis, 289 kg ha^−1^ of 17–17–17 (N–P–K) (Chouteau Lime Co., Pryor, OK) was applied pre‐plant and another 107 kg ha^−1^ of 46–0–0 (N–P–K) (Chouteau Lime Co., Pryor, OK) was applied on 10 Aug. 2017 to promote growth. During the establishment phase, Permit (halosulfuron‐methyl; Gowan, Yuma, AZ) was spot sprayed to control yellow nutsedge (*Cyperus esculentus* L.) and Scimitar (lambda‐cyhalothrin; Syngenta, Greensboro, NC) to control fall armyworm [*Spodoptera frugiperda* (J.E. Smith)]. Alleys were sprayed with a 2% solution (v/v) of Roundup (glyphosate; Monsanto, St. Louis, MO) and 0.25% nonionic surfactant to prevent contamination of adjacent plots. Irrigation was applied to prevent drought stress as predicted by weather data from a nearby weather station (Oklahoma Mesonet, http://www.mesonet.org/index.php/agriculture/monitor). Hand weeding was implemented as needed.

### 2.3 Genotyping‐by‐sequencing

Leaf samples of the 109 S_1_ progenies and their parent were submitted to the University of Wisconsin‐Madison Biotechnology Center for sequencing. DNA was extracted from the leaves of each plant using a standard CTAB protocol (Doyle, 1987). Each of the 109 progeny DNA samples was replicated twice and the parent sample was replicated 35 times in 48‐plex reaction libraries. Libraries were prepared following the protocol described by Elshire et al. (2011) with minor modifications. Fifty micrograms of genomic DNA was digested with restriction enzyme 5‐bp cutter *ApeK*I (New England Biolabs, Ipswich, MA). Then the digested DNA was ligated with barcode adapters (Elshire et al., 2011) amenable to Illumina sequencing with T4 ligase (New England Biolabs, Ipswich, MA). Adapter‐ligated samples were pooled and amplified to provide library quantities amenable for sequencing. The quality and quantity of the finished libraries were assessed using the Agilent Bioanalyzer High Sensitivity Chip (Agilent Technologies, Inc., Santa Clara, CA) and Qubit dsDNA HS Assay Kit (Life Technologies, Grand Island, NY), respectively. A cluster was generated using HiSeq SR Cluster Kit v3 cBot kits (Illumina, San Diego, CA). The whole population was sequenced in three lanes on an Illumina HiSeq 2500 (Illumina, San Diego, CA) with 100 bp single‐end reads. SNP discovery was conducted using the UNEAK pipeline in TASSEL V4.3.5 with following settings: c = 5, e = 0.03, mnMAF = 0.05, mxMAF = 0.5, mnC = 0.1, mxC = 1. Raw SNPs were filtered to improve quality with the following steps: 1. SNPs with missing data were removed; 2. all redundant SNPs were removed; and 3. SNPs were removed if the parent showed ‘hh’ or ‘kk’ genotypes. For diploid species, a minimal sequencing depth of 6x is needed to ensure 98% accuracy of calling heterozygotes by assuming a binomial distribution at two alleles per locus. The remaining SNPs were filtered again to improve the confidence of correct calling with the following steps: 1. SNPs were removed if more than 10% of individual genotypes had less than 6x total reading depth; 2. the genotypes with the reading depth of only 1x and 2x were converted into missing data; and 3. SNPs with more than 5% of missing data were removed.

### 2.4 SSR markers and gel scoring

To investigate the relevance of genetic maps between *C. dactylon* and *C. transvaalensis*, SSR markers that were mapped in a *C. dactylon* genetic map (Guo et al., 2017) were genotyped in the *C. transvaalensis* S_1_ population. Two hundred and fifty‐four *C. dactylon* SSR PPs were genotyped in this population based on Guo et al. (2017). Segregation classes, hh, hk, kk were used for scoring gels (Guo et al., 2017). Briefly, for a heterozygous locus of the parent with two alleles, h and k, when only an upper band appeared in a progeny sample, it was scored as homozygous ‘hh’; when only one lower band was present, it was scored as homozygous ‘kk’; when two bands were present, the genotype was scored as heterozygous ‘hk; and the missing genotype (no band showing up) was scored as ‘‐’.

### 2.5 Linkage analysis

Linkage analysis was carried out using JoinMap 5.0 (Van Ooijen, 2006a). In JoinMap, it was reported that cross‐pollination (CP) was not appropriate for the selfed population, and the markers segregate types < hkxhk > in coupling {00} and repulsion {11} phases in both parents would cause singularity errors in QTL analysis (Dong et al., 2015). Therefore, population type F_2_ was used. The genotypes (hh, hk, kk) were converted to (a, h, b) based on the coupling (00) or repulsion (11) phase that was calculated by JoinMap. According to Dong et al. (2015), for (00) phase markers, hh > a, hk > h, kk > b; and for (11) phase markers, hh > b, hk > h, kk > a. Markers segregation ratios were calculated using Chi‐square analysis to test the goodness of fit to the expected 1:2:1 ratio. The distorted segregation markers were kept for linkage analysis as it was expected that selfing‐depression would be common in the selfed population. Van Ooijen (2006a) recommended using a starting logarithm of odds (LOD) value of 10 for a stringent test. Then we gradually decreased LOD value to regroup markers. A minimum LOD value of eight was selected because of using a smaller LOD value, markers from different linkage groups were grouped together. The regression mapping algorithm was employed with default settings except for using Kosambi as the mapping function to correct the linkage distance (Wang et al., 2015). Due to JoinMap regression algorithm not being able to handle a large number of markers in a single group, marker ordering was initially determined based on the maximum likelihood algorithm with default settings. According to the result, redundant markers located at the same positions were manually removed. Subsequently, the regression algorithm was used to re‐estimate intermarker spacing. Another dataset excluding segregation distorted markers (chi‐squared test, *P* < .05) was utilized to construct the linkage map with the same settings. Comparisons between the two linkage maps were drawn with MapChart (Voorrips, 2002).

### 2.6 Phenotypic evaluation of establishment rate and QTL analysis

The first set of visual establishment ratings on percent green cover (Cover1) was collected on 21 Aug. 2017. A 0.9 m by 0.9 m frame was used to mark the plot size to assist the percent green cover assessment. To obtain objective data, an image was taken in each plot (Cover2) on 10 Oct. 2017 with a digital camera (Canon Powershot G1X, Canon Inc., Melville, NY) mounted on a custom‐built lightbox having four compact fluorescent light bulbs as light sources (Richardson, Karcher, & Purcell, 2001). Images were analyzed by Turf Analyzer (Karcher, Purcell, Richardson, Purcell, & Kenneth, 2017). After *C. transvaalensis* plants went dormant, the third set of visual percent green cover ratings was collected on 10 November 2017 (Cover3) to quantify the first growing season total growth. All the percent green cover data were analyzed by SAS 9.4 (SAS Institute Inc., Cary, NC). A General Linear Models Procedure (PROC GLM) with split plot in time where genotype as main plot and date as subplot was utilized to conduct the analysis of variance (ANOVA) test.

Mean percent green cover data for each genotype (N = 3) were analyzed by date for QTL mapping with MapQTL 6.0 (Van Ooijen, 2006b). LOD threshold was calculated by a permutation test of 1,000 iterations at a 95% confidence level. The interval mapping (IM) method with a regression algorithm was initially selected to detect QTL. Settings for calculation options were “yes” for QTL analysis; fit dominance for F2 (IM); “1” for mapping step size; “5” for maximum number of neighboring markers; “200” for maximum number of iterations; “1.0e^−8^” for functional tolerance value; “*P* = .02” for automatic cofactor selection. Then the QTLs found in IM were further analyzed using multiple QTL model (MQM). Markers close to the peak LOD values in each LG were selected as cofactors in MQM analysis. The MQM approach was repeated until the selected cofactors consistently had the highest LOD values in each LG. A QTL graph was drawn using MapChart (Voorrips, 2002).

### 2.7 Linkage map comparison

The African bermudagrass linkage map based on OKC1163 was compared with the common bermudagrass linkage map reported by Guo et al. (2017). The *C. dactylon* SSR markers mapped on the *C. transvaalensis* linkages were used to reveal the relationship between the maps of the two species. Related linkage groups between *C. transvaalensis* and *C. dactylon* were drawn using MapChart (Voorrips, 2002).

## 3 RESULTS

### 3.1 Genotyping by sequencing and SNP calling

With the 109 progenies and the parent sequenced, a total of more than 712 million raw reads (71.2 Gbp) were obtained. The number of reads of each selfed progeny ranged from 2.1 to 7.5 million with an average of 4.7 million. The parent was sequenced 35 times, a greater depth than progeny to accurately detect the segregation of SNPs. A total of 169 million raw reads were generated from the parent and the number of reads for each replicate of the parent ranged from 2.8 to 6.8 million with an average of 4.8 million reads. After the SNP calling and the sequenced reads were trimmed to 64 bp in length, 94,909 raw SNPs were obtained. After stringent filtering, 2,026 high‐quality SNP markers were utilized for the subsequent linkage analysis.

### 3.2 SSR marker testing

Two hundred and fifty‐four *C. dactylon* SSR PPs were screened on the parent and three progenies with two replications. One hundred and twenty‐three PPs (50.4%) amplified bands representing alleles, in which 36 (29.3%) PPs were polymorphic. Those polymorphic PPs were genotyped in the whole population. Primer pair CDCA2‐177 (5′‐CACGACGTTGTAAAACGACTTGATGCACTTCCAACCAGT‐3′) and CDCA2‐178 (5′‐CGCACAGATTTGCTGATTCT‐3′) amplified each of two alleles at two loci. Totally 37 SSR markers were used for linkage map construction.

### 3.3 Linkage mapping

**FIGURE 1:**
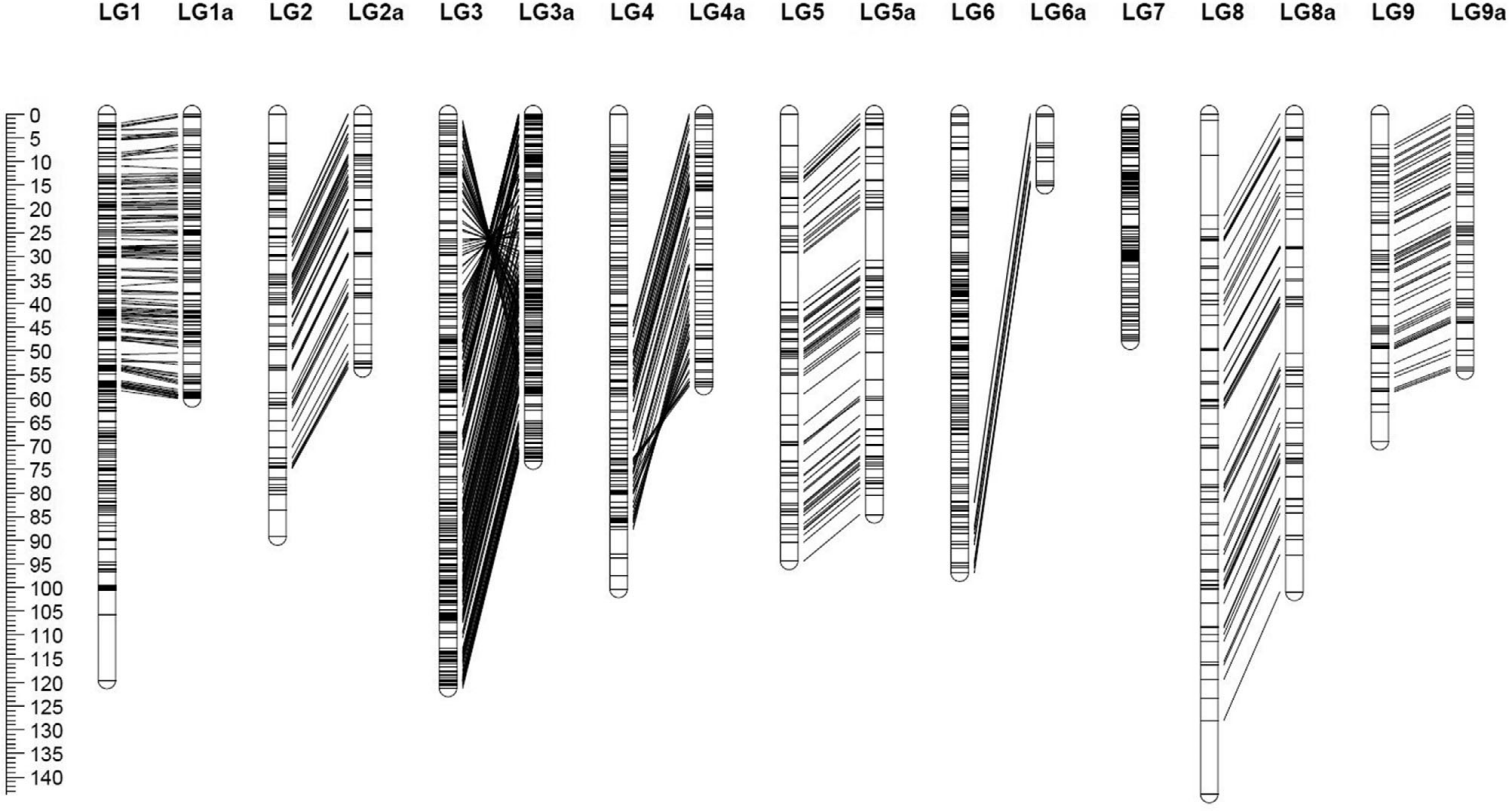
Two *Cynodon transvaalensis* linkage maps, one (LG1‐9) constructed with both undistorted and distorted markers and another (LGa1‐9) only with undistorted loci (with suffix “a” after each LG). Black lines between two LGs connect shared markers on the two maps showing how distorted loci affected the loci order. Length scale in centiMorgan (cM) on the left side

Among the 2,063 SNP and SSR markers, 1,985 (96.2%) were mapped into 9 LGs while the remaining 78 isolated markers were not able to group into any LG. Since no reference genome was available for *C. transvaalensis*, the designation of 1–9 LGs was based on the grouping numbers of JoinMap. After removing redundant markers located at the same positions in each LG calculated by maximum likelihood algorithm, 1,278 markers (1,246 SNP and 32 SSR) (Supplementary File 1) were analyzed again with the regression mapping algorithm. A summary of the LGs is given in Table 1, and graphically displayed in Figure 1. Nine LGs were generated with a total length of 882.28 cM with an average distance of 0.69 cM between neighboring markers. The number of markers in each LG ranged from 61 (LG9) to 267 (LG3) with an average of 142. The length of each LG ranged from 47.91 (LG7) to 143.60 cM (LG8). LG8 had the lowest marker density at a spacing of 2.05 cM per interval while LG7 had the highest marker density at 0.37 cM per interval. Thirty‐two of 37 (86.5%) SSR markers were mapped into eight LGs and no SSR marker was mapped into LG2 (Table 2). The number of SSR markers in LGs1, 3, 4, 5, 6, 7, 8, and 9 were 9, 7, 3, 2, 3, 1, 2, and 5, respectively. Two loci amplified by PP CDCA2‐177/178 were mapped, one on LG3 and another on LG5. One gap >15 cM was found on LG8.

**TABLE 1:** Linkage group (LG) assignment, loci numbers, LG length, marker interval, and the number of gaps in *Cynodon transvaalensis* genetic maps constructed with all markers and with only undistorted markers based on a self‐pollinated population of OKC1163

Linkage map with all markers					Linkage map with undistorted markers				
LG	Total loci	Total length cM	cM/Marker interval	Gap (>15 cM)	LG	Total loci	Total length cM	cM/Marker interval	Gap (>15 cM)
LG1	230	119.57	0.52	0	LG1a	131	60.60	0.46	0
LG2	98	89.27	0.91	0	LG2a	53	53.70	1.01	0
LG3	267	121.24	0.45	0	LG3a	256	73.18	0.29	0
LG4	157	100.33	0.64	0	LG4a	77	57.47	0.75	0
LG5	77	94.43	1.23	0	LG5a	68	84.60	1.24	0
LG6	188	96.88	0.52	0	LG6a	12	15.04	1.25	0
LG7	130	47.91	0.37	0					
LG8	70	143.60	2.05	1	LG8a	55	100.97	1.84	0
LG9	61	69.11	1.13	0	LG9a	56	54.21	0.97	0
Total	1278	882.28		1	Total	708	499.77		0
Average	142	98.03	0.69	0.11	Average	88.5	62.47	0.71	0

**TABLE 2:** Quantitative trait loci identified using interval mapping for establishment rate in a self‐pollinated population from *Cynodon transvaalensis* OKC1163

				Position of LOD peak		Phenotypic variance explained		
Dataset	LG	QTL	LOD peak	cM	Locus	%	Additive	Dominance
Cover1	LG1	*QCTER1*	5.51	60.22	TP101403	12.10	−12.38	13.61
	LG3	*QCTER2*	7.88	103.26	TP12397	17.80	−3.62	5.98
Cover2	LG3	*QCTER2*	8.33	103.07	TP64377	26.60	−14.09	17.04
Cover3	LG1	*QCTER3*	6.37	37.48	TP81783	13.40	12.31	−0.84
	LG3	*QCTER2*	12.23	103.07	TP64377	32.70	−13.84	18.40
	LG6	*QCTER4*	4.39	36.84	TP70570	8.90	24.07	28.53

*Note*: LG, linkage group; QTL, quantitative trait loci; LOD, logarithm of the odds.

Segregation distortion (*P *< .05) was observed on 569 of 1,278 (44.5%) mapped loci. Segregation distortion loci were found in all nine LGs. The percentage of segregation distortion loci in each LG ranged from 3.4% (LG3) to 100% (LG7). The distorted loci were found unevenly distributed across LGs except LG7. The majority of the distorted loci were clustered together and formed segregation distortion regions at ends of LGs (Figure 1). The distorted loci clustered in one end of LGs 1, 4, and 6 spanning more than 60, 40, and 80 cM, respectively. Among the 32 mapped SSR loci, seven (21.9%) were distorted (*P *< .05). The distorted SSR loci were mapped on LGs 1, 4, 6, 7, and 9.

To investigate how distortion segregation markers affected grouping, loci orders, and map length, a second linkage map was constructed without segregation distortion markers (Figure 1). In the above mapping analysis, all markers on LG7 were distorted in segregation. Therefore, eight LGs were created with a LOD value of eight. The number of markers in each of undistorted LGs ranged from 12 (LG6a) to 256 (LG3a). Compared with each LG in the map that consisted of distorted and undistorted loci, the removal of distorted loci did not change the grouping of undistorted markers (Figure 1). The total length of the undistorted linkage map was 499.77 cM with an average distance of 0.71 cM per marker interval (Table 1). This linkage map with undistorted markers was 43.4% shorter than the map that included both distorted and undistorted loci. No gap larger than 15 cM was observed on the LGs1a‐9a.

### 3.4 Establishment rate (ER) and QTL analysis

Three sets of percent green cover data were taken to quantify the establishment rate for each of the progeny and the parent. The ANOVA results indicated a significant evaluation date by genotype effect (Supplementary File 2 and Table S1). The means and associated standard deviations for Cover1, Cover2, and Cover3 in the progeny population were 18.14 ± 11.93, 44.77 ± 32.74, and 58.15 ± 27.06, respectively (Supplementary File 3). The results of the percent green cover of the parent in Cover1, Cover2, and Cover3 were 22.67 ± 13.65, 66.87 ± 36.84, and 85.00 ± 15.00, respectively. Genotype means were highly and positively correlated (0.83 to 0.93) across evaluation dates.

**FIGURE 2:**
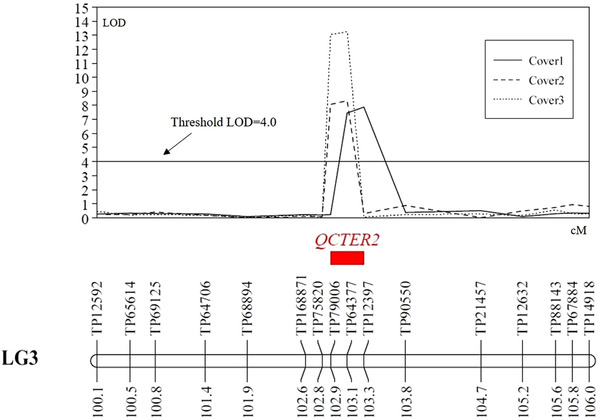
A major quantitative trait locus, *QCTER2* associated with African bermudagrass establishment rate identified at three different time points located at the region on linkage group 3, where genetic distances in centiMorgan (cM) are given on the lower side of the map while SNP marker designations are shown on the upper side of the map bar. The horizontal line with a LOD value of 4.0 indicates the 95% significant threshold for QTL detection

The LOD threshold calculated for detecting ER QTL was four in the QTL analysis. Four QTLs for ER, designated *QCTER1‐4*, were identified in the progeny population. They were located on LGs1, 3, and 6, which explained 8.9 to 32.7% of the total phenotypic variation (Table 2). Among these QTLs, *QCTER2*, with the peak LOD values at positions 103.26 and 103.07 cM on LG3 was detected in all three datasets, explaining 17.8–32.7% of the total phenotypic variation (Figure 2). Other three QTLs were identified in only one dataset each (Table 2). The major QTL, *QCTER2* was closely linked to SNP markers, TP64377/TP12397 on LG3. The intra‐locus effects of the QTLs linked markers are given in Table 2. The means and associated standard deviations for the percent green cover of the QTLs linked markers are given in Table 3.

**TABLE 3:** Means and standard deviations of three percent green cover data collections in an S_1_ population of two SNP markers that are closely linked to a major QTL, *QCTER2*

Marker	Genotype	Cover1 %	Cover2 %	Cover3 %
TP64377	A (kk)	9.94 ± 5.38b ^a^	21.24 ± 16.97b	35.22 ± 18.83b
	H (hk)	20.16 ± 6.89a	52.75 ± 20.60a	65.77 ± 16.33a
	B (hh)	19.25 ± 9.46a	48.29 ± 25.34a	59.76 ± 22.08a
TP12397	A (hh)	9.94 ± 5.38b	21.24 ± 16.97b	35.22 ± 18.83b
	H (hk)	20.46 ± 7.23a	53.32 ± 21.75a	66.04 ± 17.43a
	B (kk)	20.32 ± 10.88a	50.19 ± 25.40a	61.90 ± 22.02a

^a^ Values with a common letter within a column by marker are not significantly different at *P <* .05.

### 3.5 Linkage map comparison

The incorporation of *C. dactylon* SSR markers in this *C. transvaalensis* linkage map allowed for an investigation of the genomic relationship between the two species. Among *C. transvaalensis* LGs, except LG2, each had at least one SSR marker. Each of *C. transvaalensis* LGs5, 6, 7, and 8 was connected to one *C. dactylon* LG and each of *C. transvaalensis* LGs 1 and 4 corresponded to two *C. dactylon* LGs. The remaining two *C. transvaalensis* LGs (LGs3 and 9) were each bridged to three *C. dactylon* LGs ((Supplementary File 2 and Figures S1 to S8).

## 4 DISCUSSION

### 4.1 Selfed mapping population

Although *Cynodon* spp. can be easily propagated vegetatively by rhizomes or stolons, most *Cynodon* spp. plants still have the ability to sexually reproduce by seed (Richardson et al., 1978). Kneebone (1967) reported a low percent seed set produced by self‐pollination with bagging, suggesting the mode of sexual reproduction in the bermudagrass taxon is through a high degree of cross‐pollination with relatively high self‐incompatibility (Richardson et al., 1978). However, *C. transvaalensis* genotype OKC1163 was able to produce a large amount of selfed seed, from which an S_1_ population was developed for the linkage map construction in this study. Since the male and female gametes were produced on the same parent, only one genetic linkage map was constructed. Several other studies used S_1_ populations to construct genetic linkage maps for outcrossing species such as *C. dactylon* (Guo et al., 2017), sugarcane (*Saccharum* spp.) (Andru, Pan, Thongthawee, Burner, & Kimbeng, 2011), and switchgrass (Dong et al., 2015; Liu, Wu, Wang, & Samuels, 2012). Population size is an important factor in linkage map construction which affects the linkage map accuracy. *Cynodon transvaalensis* linkage maps reported by Bethel et al. (2006) and Harris‐Shultz et al. (2010) included 113 and 118 gametes in linkage analysis. In this study, 218 gametes of 109 S_1_ progeny were used to calculate genetic distances to form the *C. transvaalensis* linkage map. More gametes in this linkage analysis provided a higher level of accuracy in calculating the recombination frequency.

### 4.2 Linkage map

Using the GBS approach, 109 selfed progenies and the parent OKC1163 were sequenced and more than 94,000 SNP markers were generated. Eventually, 1,246 SNP and 32 SSR markers were used in the construction of this map in *C. transvaalensis*. To our knowledge, this map was the first high‐density genetic linkage map in the species. Bethel et al. (2006) constructed a framework linkage map of *C. transvaalensis* using an interspecific hybrid population between *C. dactylon* T89 × *C. transvaalensis* T574 based on single‐dose restriction fragments (SDRFs). The T574 *C. transvaalensis* map was constructed with 77 markers on 18 LGs with an average marker spacing of 16.5 cM (Bethel et al., 2006). Harris‐Shultz et al. (2010) reported a T574 map based on 36 single‐dose amplified fragments (SDAFs) spanning 311.1 cM. Khanal et al. (2017) enriched SSR markers to the SDRF framework of Bethel et al. (2006), producing eleven LGs spanning 1,184.7 cM with 52 SSR and 73 SDRF markers. Compared to the total markers used in the maps by Bethel et al. (2006), Harris‐Shultz et al. (2010) and Khanal et al. (2017), 1,278 markers, more than 10 times of previously mapped, were included in the OKC1163 map.

*C. transvaalensis* can easily cross with *C. dactylon*, producing interspecific hybrids. To investigate the relationship between *C. transvaalensis* and *C. dactylon* LGs, we tested 244 *C. dactylon* SSR PPs, in which 36 PPs were polymorphic and one PP was multiallelic. The multiallelic PP CDCA2‐177/178 mapped to LG3 and LG5 may indicate paralogous loci in *C. transvaalensis* genome. None of the published *C. transvaalensis* maps has reported primer pairs that were able to amplify two loci. However, in tetraploid species, Guo et al. (2017) reported four multiallelic PPs, which identified three homoelogous LGs in *C. dactylon*.

Each *C. transvaalensis* LG corresponded to at least one *C. dactylon* LG as compared to the linkage map by Guo et al. (2017) (Supplementary Figures S1 to S8). Khanal et al. (2017) also reported that most *C. transvaalensis* LGs corresponded to two *C. dactylon* LGs and some *C. transvaalensis* LGs corresponded to either one or three *C. dactylon* LGs. *Cynodon dactylon* was considered an allopolyploid tetraploid species (Fang et al., 2020; Gong, Xue, Zhang, & Wang, 2013; Guo, Wu, Anderson, Moss, & Zhu, 2015). More research is needed to definitively characterize the genomic relationship between the two closely related species. Once whole genome sequence is available in one or both species, it is feasible to fully establish the relationship.

### 4.3 Segregation distortion

Segregation distortion is a common phenomenon in cross‐pollinated species like *C. dactylon* (Guo et al., 2017; Khanal et al., 2017), switchgrass (Fiedler et al., 2015; Liu et al., 2012), and zoysiagrass (Huang et al., 2016; Wang et al., 2015). Linkage maps constructed with segregation distorted markers may lead to incorrect marker orders due to spurious linkages (Cloutier, Cappadocia, & Landry, 1997). In this self‐pollinated *C. transvaalensis* population, 44.5% mapped markers were segregation distorted. The segregation distortion rate in this population was slightly higher compared to the segregation distortion rate of the self‐pollinated *C. dactylon* population reported by Guo et al. (2017). Khanal et al. (2017) reported 12.1% of distorted loci in the *C. transvaalensis* parental map that derived from a *C. dactylon* × *C. transvaalensis* population. Our results are consistent with the higher tendency for segregation distortion due to selfing that theoretically generates homozygous loci of lethal alleles in the self‐pollinated population as compared with the cross‐pollinated population (Guo et al., 2017; Liu et al., 2012).

Most distorted markers were clustered near the central part or ends of the LGs. In LGs1, 2 and 4 of this map, most distorted markers were clustered at one half of LGs. But the distorted markers in LGs5 and 9 were scattered at both ends. All the markers in LG7 were distorted. Guo et al. (2017) also reported that the markers in two *C. dactylon* LGs were all distorted. We found that the total length of the linkage map with distorted markers was over 90% longer than the map that only included undistorted markers. Fiedler et al. (2015) and Huang et al. (2016) reported 23% and around 10% increased length when adding distorted markers into switchgrass and zoysiagrass linkage maps based on cross‐pollinated populations, respectively.

When comparing the shared markers and their orders between the distorted‐ and undistorted‐marker linkage maps, we observed rearrangements on LGs1, 2, 3, 4, and 5. In LGs2 and 5, reversed marker orders were observed between neighboring markers (Figure 1). LG1 experienced more extensive marker rearrangements, some markers were relocated to a new position a few markers away. On LGs3 and 4, marker orders were inverted at one end of each. If distorted markers were clustered on one or two ends of an LG, the marker orders appeared not to be affected by adding distorted markers. However, if distorted markers were located in the middle of an LG, marker orders were likely to be disrupted. This suggested that segregation distortion markers affected the linkage analysis, yet differently upon their locations in an LG. However, it is still recommended to keep distorted markers in linkage analysis to better understand LGs (Van Ooijen, 2006a). Segregation distortion marker clustering regions have not been analyzed in the *C. transvaalensis* genome before. The clustering regions of distorted markers usually contain lethal genes or incompatibility genes (Lefebvre, Palloix, Caranta, & Pochard, 1995). Therefore, having the knowledge of distorted loci are important in conventional breeding since distorted loci are important factors in sexual reproduction (i.e., seed set) (Shinozuka, Cogan, Smith, Spangenberg, & Forster, 2010).

### 4.4 QTLs of establishment rate in *C. transvaalensis*

This was the first QTL study of establishment rate in *C. transvaalensis* based on a high‐density linkage map. The results clearly showed that the *C. transvaalensis* ER was controlled by multiple loci. In total, four QTLs were identified in this study. The QTL on LG3, *QCTER2*, with peak LOD value near TP64377/TP12397 were detected consistently across all three datasets. The consistency of QTLs at the same location in a LG is a good indication of the reliability of the results. One QTL, *QCTER1* with peak LOD value at 60.22 cM on LG1 was only detected in Cover1, another QTL, *QCTER3* at 37.48 cM on LG1 only detected in Cover3, and the QTL, *QCTER4* at 36.84 cM on LG6 was only detected in Cover3, suggest theses QTLs may play roles in different growth and developmental stages. Besides differences in growth and development stages, unrepeatable QTLs were likely the result of gene by environment interaction, which may be affected by environmental conditions (i.e., soil type, water, light, and temperature) (Johnson, Haggard, & St. Clair, 2012). Guo et al. (2017) identified five QTLs associated with *C. dactylon* ER. They reported one QTL on *C. dactylon* LG18 to be reliable. In this study, we identified *QCTER4* in *C. transvaalensis* LG6, orthologous to *C. dactylon* LG18. In addition, the orthologous *C. transvaalensis* LG3 and *C. dactylon* LG16, both carried QTLs associated with ER. The QTL analysis in this study was based on a high‐density genetic linkage map, which showed a greater detection resolution and the QTLs were mapped to smaller regions compared to the QTL mapping by Guo et al. (2017). Markers closely linked to QTLs provide a valuable tool in marker‐assisted selection for ER. Once the whole genome sequence of *C. transvaalensis* is available, the sequences of these tightly linked SNP markers can anchor the physical locations of QTLs on *C. transvaalensis* chromosomes and be converted to breeder friendly PCR‐based markers that are specific for those respective QTLs. Those markers will be useful in marker‐assisted selection for developing fast ER bermudagrass hybrid cultivars.

### AUTHOR CONTRIBUTIONS

SHY conducted the experiments, analyzed the data, and wrote the manuscript as a part of his PhD research. TLF and HXD helped in lab work and data analyses. LLY, DLM, JQM, and CHF served as SHY's committee members, guided him through his research work and revised the manuscript before submission. YQW conceived the project, created the population, served as SHY's major advisor, and revised the manuscript before submission.

## CONFLICT OF INTEREST

The authors declare they have no conflict of interest.

## Supporting information



SUPPLEMENTARY MATERIAL

SUPPLEMENTARY MATERIAL

SUPPLEMENTARY MATERIAL

SUPPLEMENTARY MATERIAL

## References

[tpg220073-bib-0001] Andru, S. , Pan, Y. B. , Thongthawee, S. , Burner, D. M. , & Kimbeng, C. A. (2011). Genetic analysis of the sugarcane (*Saccharum* spp.) cultivar ’LCP 85–384’. I. Linkage mapping using AFLP, SSR, and TRAP markers. Theoretical and Applied Genetics, 123, 77–93. 21472411 10.1007/s00122-011-1568-x

[tpg220073-bib-0002] Bethel, C. M. , Sciara, E. B. , Estill, J. C. , Bowers, J. E. , Hanna, W. W. , & Paterson, A. H. (2006). A framework linkage map of bermudagrass (*Cynodon dactylon* × *transvaalensis*) based on single‐dose restriction fragments. Theoretical and Applied Genetics, 112, 727–737. 16395568 10.1007/s00122-005-0177-y

[tpg220073-bib-0003] Burton, G. W. (1991). A history of turf research at Tifton. U.S. Golf Association Green Section Record 29:12–14.

[tpg220073-bib-0004] Burton, G. W. , & Hart, R. H. (1967). Use of self‐incompatibility to produce commercial seed‐propagated F_1_ bermudagrass hybrids. Crop Science, 7, 524–527. 10.2135/cropsci1967.0011183X000700050035x

[tpg220073-bib-0005] Cloutier, S. , Cappadocia, M. , & Landry, B. S. (1997). Analysis of RFLP mapping inaccuracy in *Brassica napus* L. Theoretical and Applied Genetics, 95, 83–91. 10.1007/BF00223890 24169967

[tpg220073-bib-0006] Dong, H. X. , Thames, S. , Liu, L. L. , Smith, M. W. , Yan, L. L. , & Wu, Y. Q. (2015). QTL mapping for reproductive maturity in lowland switchgrass populations. BioEnergy Research, 8, 1925–1937.

[tpg220073-bib-0007] Dong, H. X. , Liu, S. Y. , Clark, L. V. , Sharma, S. , Gifford, J. M. , Juvik, J. A. … Sacks, E. J. (2018). Genetic mapping of biomass yield in three interconnected Miscanthus populations. GCB Bioenergy, 10, 165–185.

[tpg220073-bib-0008] Doyle, J. J. (1987). A rapid DNA isolation procedure for small quantities of fresh leaf tissue. Phytochemical Bulletin, 19, 11–15.

[tpg220073-bib-0009] Elshire, R. J. , Glaubitz, J. C. , Sun, Q. , Poland, J. A. , Kawamoto, K. , Buckler, E. S. … Mitchell, S. E. (2011). A robust, simple genotyping‐by‐sequencing (GBS) approach for high diversity species. Plos One, 6, e19379. 21573248 10.1371/journal.pone.0019379 PMC3087801

[tpg220073-bib-0010] Fang, T. L. , Wu, Y. Q. , Makaju, S. , Tribble, T. , Martin, D. L. , & Moss, J. Q. (2015). Identification of turf bermudagrasses on the Oklahoma State University baseball field and three experimental clones as revealed with simple sequence repeat markers. HortTechnology, 25, 714–724.

[tpg220073-bib-0011] Fang, T. L. , Dong, H. X. , Yu, S. , Moss, J. Q. , Fontanier, C. H. , Martin, D. L. , … Wu, Y. Q. (2020). Sequence‐based genetic mapping of *Cynodon dactylon* Pers. reveals new insights into genome evolution in Poaceae. Communication Biology, 3, 1–10. 10.1038/s42003-020-1086-y PMC7347563 32647329

[tpg220073-bib-0012] Fiedler, J. D. , Lanzatella, C. , Okada, M. , Jenkins, J. , Schmutz, J. , & Tobias, C. M. (2015). High‐density single nucleotide polymorphism linkage maps of lowland switchgrass using genotyping‐by‐sequencing. Plant Genome, 8, 1–14. 10.3835/plantgenome2014.10.0065 33228324

[tpg220073-bib-0013] Forbes, I. , & Burton, G. W. (1963). Chromosome numbers and meiosis in some *Cynodon* species and hybrids. Crop Science, 3, 75–79. 10.2135/cropsci1963.0011183X000300010023x

[tpg220073-bib-0014] Gong, Z. , Xue, C. , Zhang, M. , & Wang, M. (2013). Distribution of rDNA loci and genome differentiation in tetraploid *Cynodon* . Indian Journal of Genetics and Plant Breeding, 73, 459–461.

[tpg220073-bib-0015] Guo, Y. W. , Wu, Y. Q. , Anderson, J. A. , Moss, J. Q. , & Zhu, L. (2015). Disomic inheritance and segregation distortion of SSR markers in two populations of *Cynodon dactylon* (L.) Pers. var. *dactylon* . Plos One, 10, e0136332. 26295707 10.1371/journal.pone.0136332 PMC4546580

[tpg220073-bib-0016] Guo, Y. W. , Wu, Y. Q. , Anderson, J. A. , Moss, J. Q. , Zhu, L. , & Fu, J. M. (2017). SSR marker development, linkage mapping, and QTL analysis for establishment rate in common bermudagrass. Plant Genome, 10, 1–11. 10.3835/plantgenome2016.07.0074 28464062

[tpg220073-bib-0017] Harlan, J. R. , de Wet, J. M. J. , & Rawal, K. M. (1970a). Geographic distribution of the species of *Cynodon* L.C. Rich (*Gramineae*). East African Agricultural and Forestry Journal, 36, 220–226.

[tpg220073-bib-0018] Harlan, J. R. , de Wet, J. M. J. , Huffine, W. W. , & Deakin, J. R. (1970b). A guide to the species of *Cynodon* (Gramineae). Oklahoma State University Agricultural Experiment Station, B‐673, 37–37.

[tpg220073-bib-0019] Harris‐Shultz, K. R. , Schwartz, B. M. , Hanna, W. W. , & Brady, J. A. (2010). Development, linkage mapping, and use of microsatellites in bermudagrass. Journal of the American Society for Horticultural Science, 135, 511–520.

[tpg220073-bib-0020] Holloway, H. M. P. , Yu, X. W. , Dunne, J. C. , Schwartz, B. M. , Patton, A. J. , Arellano, C. , & Milla‐Lewis, S. R. (2018). A SNP‐based high‐density linkage map of zoysiagrass (*Zoysia japonica* Steud.) and its use for the identification of QTL associated with winter hardiness. Molecular Breeding, 38, 10–24.

[tpg220073-bib-0021] Huang, X. E. , Wang, F. F. , Singh, R. , Reinert, J. A. , Engelke, M. C. , Genovesi, A. D. , … Yu, Q. (2016). Construction of high‐resolution genetic maps of *Zoysia matrella* (L.) Merrill and applications to comparative genomic analysis and QTL mapping of resistance to fall armyworm. BMC Genomics, 17, 562–578. 27501690 10.1186/s12864-016-2969-7 PMC4977732

[tpg220073-bib-0022] Juska, F. V. , & Hanson, A. A. (1964). Evaluation of bermudagrass varieties for general purpose turf. USDA‐ARS, Agric. Handbook No. 270. Washington, DC: USDA.

[tpg220073-bib-0023] Johnson, E. B. , Haggard, J. E. , & St. Clair, D. A. (2012). Fractionation, stability, and isolate‐specificity of QTL for resistance to *Phytophthora infestans* in cultivated tomato (*Solanum lycopersicum*). G3: Genes, Genomes, Genetics, 2, 1145–1159. 23050225 10.1534/g3.112.003459 PMC3464107

[tpg220073-bib-0024] Karcher, D. E. , Purcell, C. J. , Richardson, M. D. , Purcell, L. C. , & Kenneth, W. H. (2017). New Java program to rapidly quantify several turfgrass parameters from digital images. CSSA Oral presentation. Retrieved from https://scisoc.confex.com/crops/2017am/webprogram/Paper109313.html

[tpg220073-bib-0025] Kenworthy, K. E. , Taliaferro, C. M. , Carver, B. F. , Martin, D. L. , Anderson, J. A. , & Bell, G. E. (2006). Genetic variation in *Cynodon transvaalensis* Burtt‐Davy. Crop Science, 46, 2376–2381. 10.2135/cropsci2006.02.0075

[tpg220073-bib-0026] Khanal, S. , Kim, C. , Auckland, S. A. , Rainville, L. K. , Adhikari, J. , Schwartz, B. M. , & Paterson, A. H. (2017). SSR‐enriched genetic linkage maps of bermudagrass (*Cynodon dactylon* × *transvaalensis*), and their comparison with allied plant genomes. Theoretical and Applied Genetics, 130, 819–839. 28168408 10.1007/s00122-017-2854-z

[tpg220073-bib-0027] Kneebone, W. R. (1967). Breeding seeded bermudagrass; unique Arizona research problem. Progressive Agriculture in Arizona, 19, 4–5.

[tpg220073-bib-0028] Lefebvre, V. , Palloix, A. , Caranta, C. , & Pochard, E. (1995). Construction of an intraspecific integrated linkage map of pepper using molecular markers and doubled‐haploid progenies. Genome, 38, 112–121. 18470157 10.1139/g95-014

[tpg220073-bib-0029] Liu, L. L. , Wu, Y. Q. , Wang, Y. W. , & Samuels, T. (2012). A high‐density simple sequence repeat‐based genetic linkage map of switchgrass. G3: Genes, Genomes, Genetics, 2, 357–370. 22413090 10.1534/g3.111.001503 PMC3291506

[tpg220073-bib-0030] Liu, S. Y. , Clark, L. V. , Swaminathan, K. , Gifford, J. M. , Juvik, J. A. , & Sacks, E. J. (2016). High‐density genetic map of *Miscanthus sinensis* reveals inheritance of zebra stripe. GCB Bioenergy, 8, 616–630.

[tpg220073-bib-0031] Mammadov, J. , Aggarwal, R. , Buyyarapu, R. , & Kumpatla, S. (2012). SNP markers and their impact on plant breeding. International Journal of Plant Genomics, 2012. 10.1155/2012/728398 PMC3536327 23316221

[tpg220073-bib-0032] Nalini, E. , Jawali, N. , & Bhagwat, S. (2004). A simple method for isolation of DNA from plants suitable for long term storage and DNA marker analysis. BARC Newsletter, 249, 208–214.

[tpg220073-bib-0033] National Turfgrass Evaluation Program (NTEP) . (2017). Final Report NTEP No. 18‐14. 2013 National Bermudagrass Test. Retrieved from http://www.ntep.org/reports/bg13/bg13_18-14f/bg13_18-14f.htm

[tpg220073-bib-0034] Richardson, M. D. , Karcher, D. E. , & Purcell, L. C. (2001). Quantifying turfgrass cover using digital image analysis. Crop Science, 41, 1884–1881. 10.2135/cropsci2001.1884

[tpg220073-bib-0035] Richardson, W. L. , Taliaferro, C. M. , & Abring, R. M. (1978). Fertility of eight bermudagrass clones and open‐pollinated progeny from them. Crop Science, 18, 332–334. 10.2135/cropsci1978.0011183X001800020035x

[tpg220073-bib-0036] Schwartz, B. M. , Hanna, W. W. , Baxter, L. L. , Raymer, P. L. , Waltz, P. C. , Kowalewski, A. R. , … Milla‐Lewis, S. R. (2018). ‘DT‐1’, a drought‐tolerant triploid turf bermudagrass. Hortscience, 53, 1711–1714.

[tpg220073-bib-0037] Shinozuka, H. , Cogan, N. O. I. , Smith, K. F. , Spangenberg, G. C. , & Forster, J. W. (2010). Fine‐scale comparative genetic and physical mapping supports map‐based cloning strategies for the self‐incompatibility loci of perennial ryegrass (*Lolium perenne* L.). Plant Molecular Biology, 72, 343–355. 19943086 10.1007/s11103-009-9574-y

[tpg220073-bib-0038] Taliaferro, C. M. (1992). Out of Africa—A new look at “African” bermudagrass. *U.S. Golf Association Green Section Record*, 30, 10–12.

[tpg220073-bib-0039] Taliaferro, C. M. , & Lamle, J. T. (1997). Use of flow cytometry to estimate ploidy level in *Cynodon* species. International Turfgrass Society Research Journal, 8, 385–392.

[tpg220073-bib-0040] Taliaferro, C. M. , Martin, D. L. , Anderson, J. A. , & Anderson, M. P. (2006). Patriot turf bermudagrass. USPP 16,801. Washington, DC: U.S. Patent and Trademark Office.

[tpg220073-bib-0041] Tan, C. C. , Wu, Y. Q. , Taliaferro, C. M. , Anderson, M. P. , Tauer, C. , & Samuels, T. (2012). Development of simple sequence repeat markers for bermudagrass from its expressed sequence tag sequences and preexisting sorghum SSR markers. Molecular Breeding, 29, 23–30.

[tpg220073-bib-0042] Van Ooijen, J. W. (2006a). JoinMap 5: Software for the calculation of genetic linkage maps in experimental populations. B. V. Kyazma (Ed.). Wageningen: the Netherlands.

[tpg220073-bib-0043] Van Ooijen, J. W. (2006b). MapQTL 6: Software for the mapping of quantitative trait loci in experimental populations of diploid species. B. V. Kyazma (Ed.). Wageningen: the Netherlands.

[tpg220073-bib-0044] Voorrips, R. E. (2002). MapChart: Software for the graphical presentation of linkage maps and QTLs. Journal of Heredity, 93, 77–78. 12011185 10.1093/jhered/93.1.77

[tpg220073-bib-0045] Wang, F. F. , Singh, R. , Genovesi, A. D. , Wai, C. M. , Huang, X. E. , Chandra, A., & Yu, Q. (2015). Sequence‐tagged high‐density genetic maps of *Zoysia japonica* provide insights into genome evolution in Chloridoideae. Plant Journal, 82, 744–757. 10.1111/tpj.12842 25846381

[tpg220073-bib-0046] Wu, Y. Q. , Taliaferro, C. M. , Bai, G. H. , & Anderson, M. P. (2005). Genetic diversity of *Cynodon transvaalensis* Burtt‐Davy and its relatedness to hexaploid *C. dactylon* (L.) Pers. as indicated by AFLP markers. Crop Science, 45, 848–853. 10.2135/cropsci2003.913

[tpg220073-bib-0047] Wu, Y. Q. , Martin, D. L. , Taliaferro, C. M. , Anderson, J. A. , & Moss, J. Q. (2013). Northbridge turf bermudagrass. USPP 24,116. Washington, DC: U.S. Patent and Trademark Office.

[tpg220073-bib-0048] Wu, Y. Q. , Martin, D. L. , Taliaferro, C. M. , Anderson, J. A. , & Moss, J. Q. (2014). Latitude 36 turf bermudagrass. USPP 24,271. Washington, DC: U.S. Patent and Trademark Office.

[tpg220073-bib-0049] Wu, Y. Q. , Martin, D. L. , Moss, J. Q. , Walker, N. R. , & Fontanier, C. H. (2019). Bermudagrass plant named ‘OKC 1131’. USPP 2019/0364715 P1. Washington, DC: U.S. Patent and Trademark Office.

[tpg220073-bib-0050] Yu, X. W. , Kimball, J. A. , & Milla‐Lewis, S. R. (2018). High density genetic maps of St. Augustinegrass and applications to comparative genomic analysis and QTL mapping for turf quality traits. BMC Plant Biology, 18, 346–357. 30541451 10.1186/s12870-018-1554-4 PMC6292074

[tpg220073-bib-0051] Zhang, L. H. , Ozias‐Akins, P. , Kochert, G. , Kresovich, S. , Dean, R. , & Hanna, W. W. (1999). Differentiation of bermudagrass (*Cynodon* spp.) genotypes by AFLP analyses. Theoretical and Applied Genetics, 98, 895–902.

