# Supplementary material for: Genetic and QTL mapping in African bermudagrass
Source: Plant Genome. 2021 Mar 3;14(1):e20073. doi: 10.1002/tpg2.20073 (PMC12807324; doi:10.1002/tpg2.20073)
Supplement: Supplementary file 1 [file TPG2-14-e20073-s004.pdf]

Table S1. Analysis of variance for the effects of entry, date, block and their interaction, on establishment rate in *Cynodon transvaalensis*

Source	df	SS	MS	F
Entry	108	325870.8	3017.3	27.8***
Block	2	16552.7	8276.4	76.1***
Entry*Block	216	165221.7	765.0	7.0***
Date	2	267690.6	133845.3	1231.4***
Entry*Date	216	59620.0	276.0	2.5***
Date*Block*(Entry)	421	45759.6	108.7	
Total	965			

***, significant at $P < 0.0001$

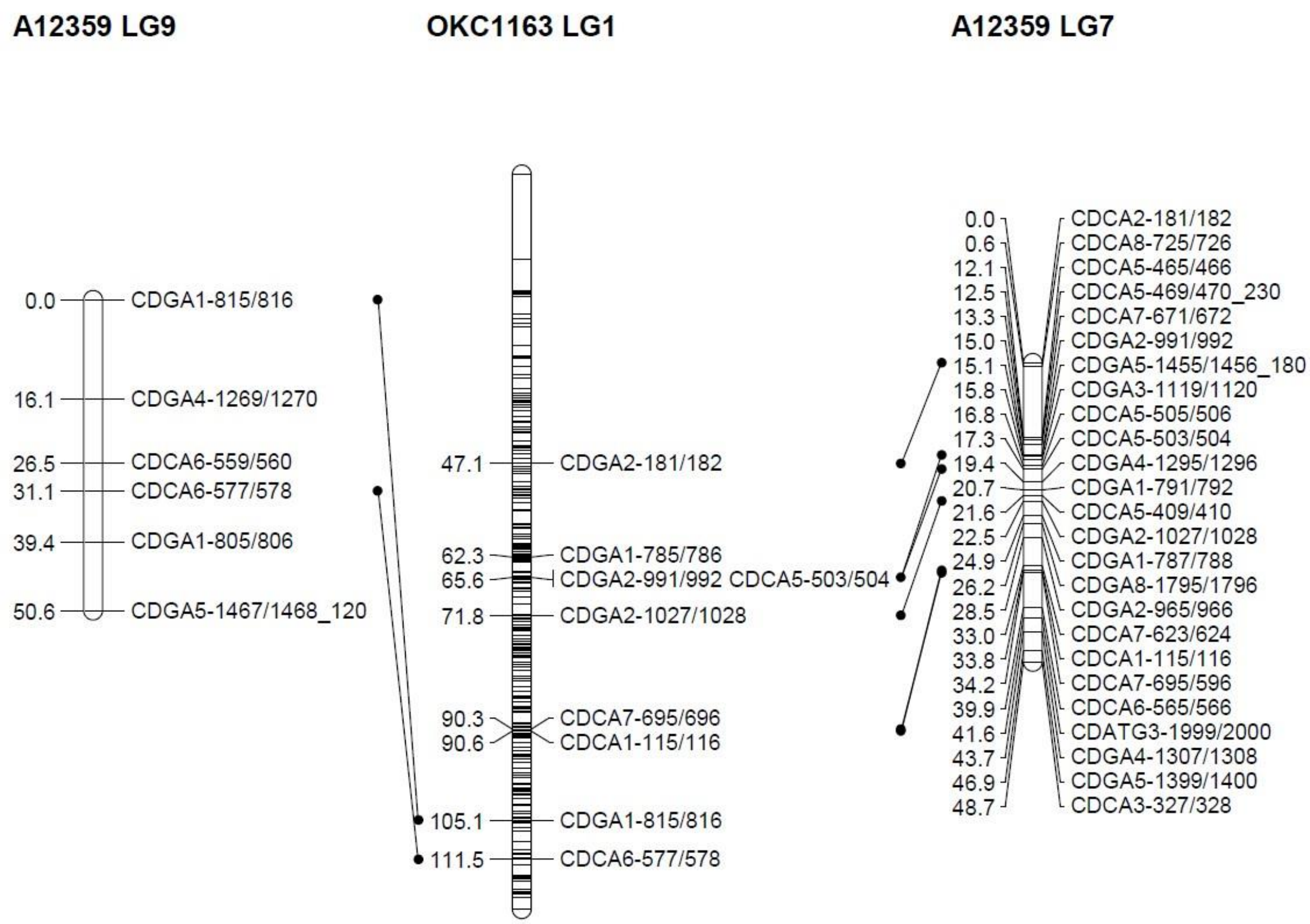

Fig. S1. *Cynodon transvaalensis* LG1 corresponding to *Cynodon dactylon* LGs7 and 9 (Guo *et al.*, 2017).

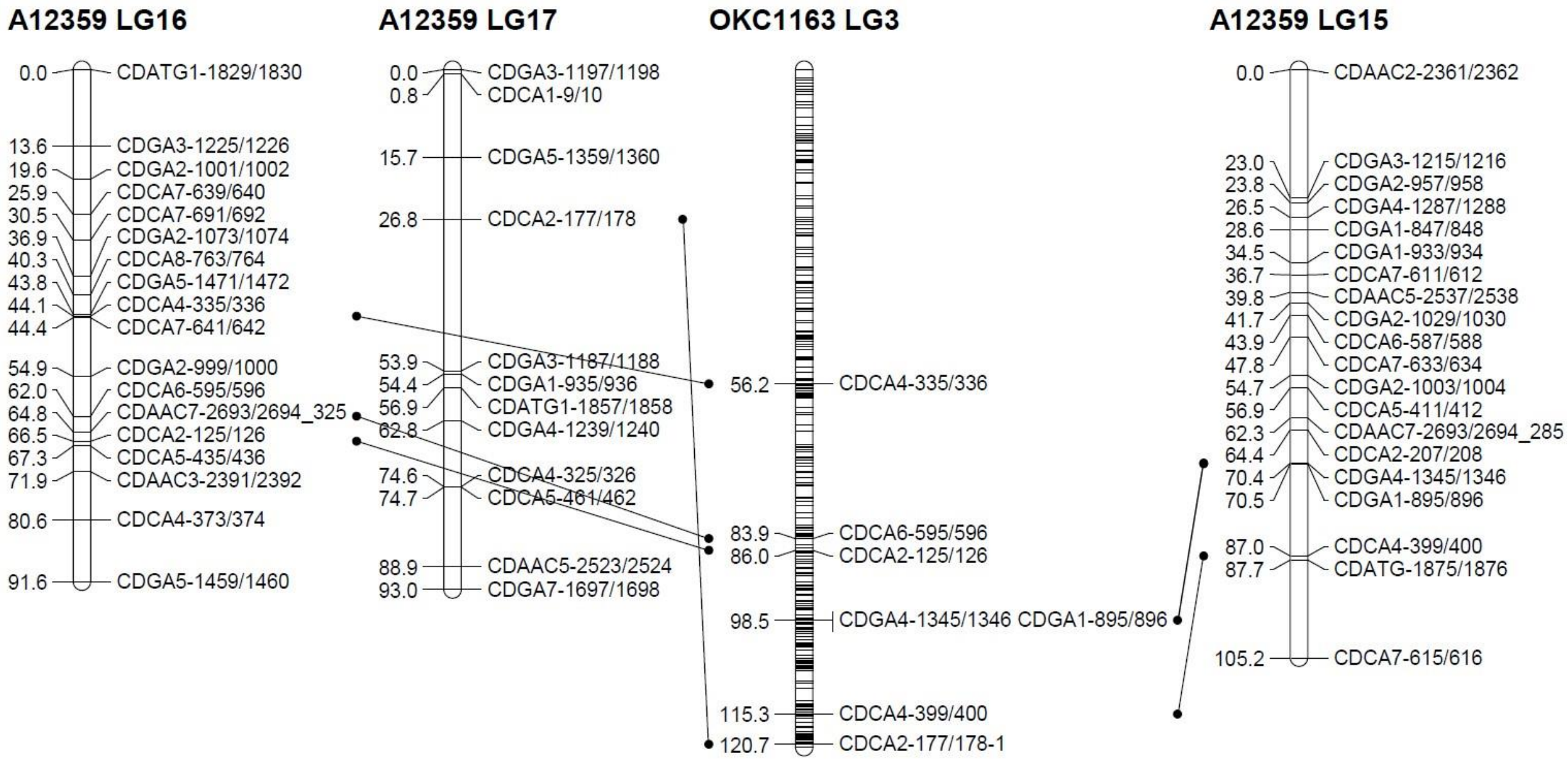

Fig. S2. *Cynodon transvaalensis* LG3 corresponding to *Cynodon dactylon* LGs15, 16, and 17 (Guo *et al*., 2017).

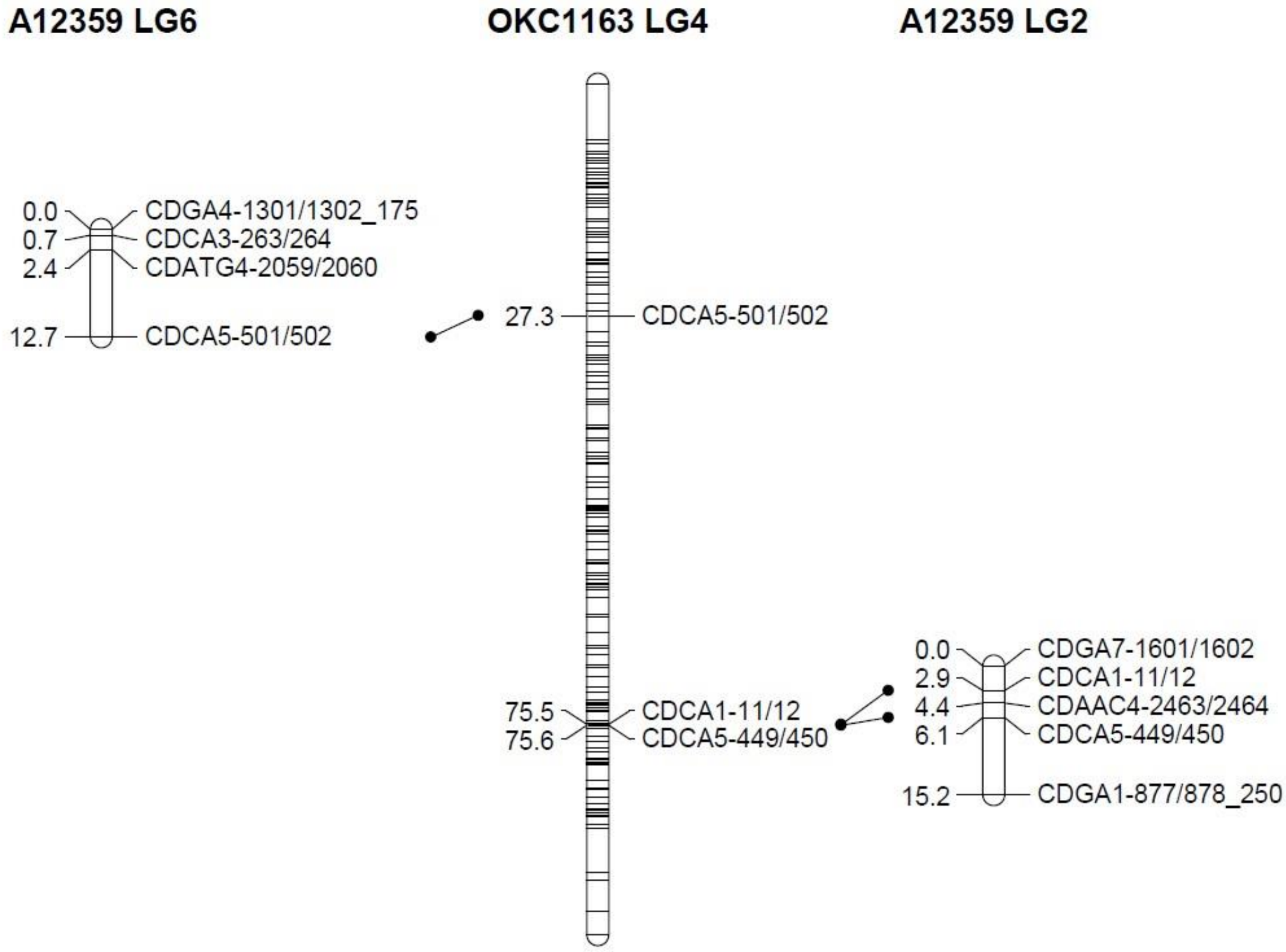

Fig. S3. *Cynodon transvaalensis* LG4 corresponding to *Cynodon dactylon* LGs2 and 6 (Guo *et al*., 2017).

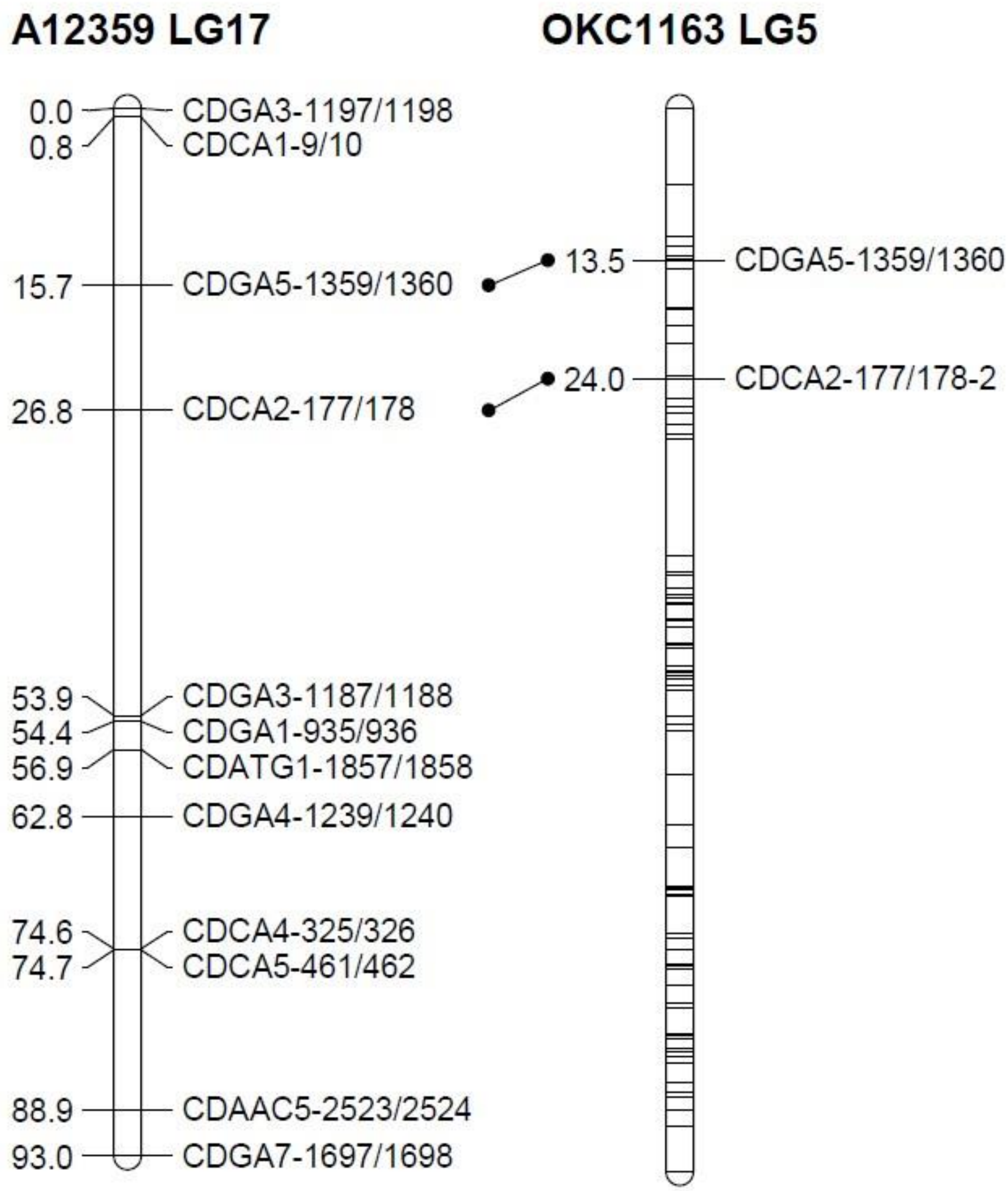

Fig. S4. *Cynodon transvaalensis* LG5 corresponding to *Cynodon dactylon* LG17 (Guo *et al*., 2017).

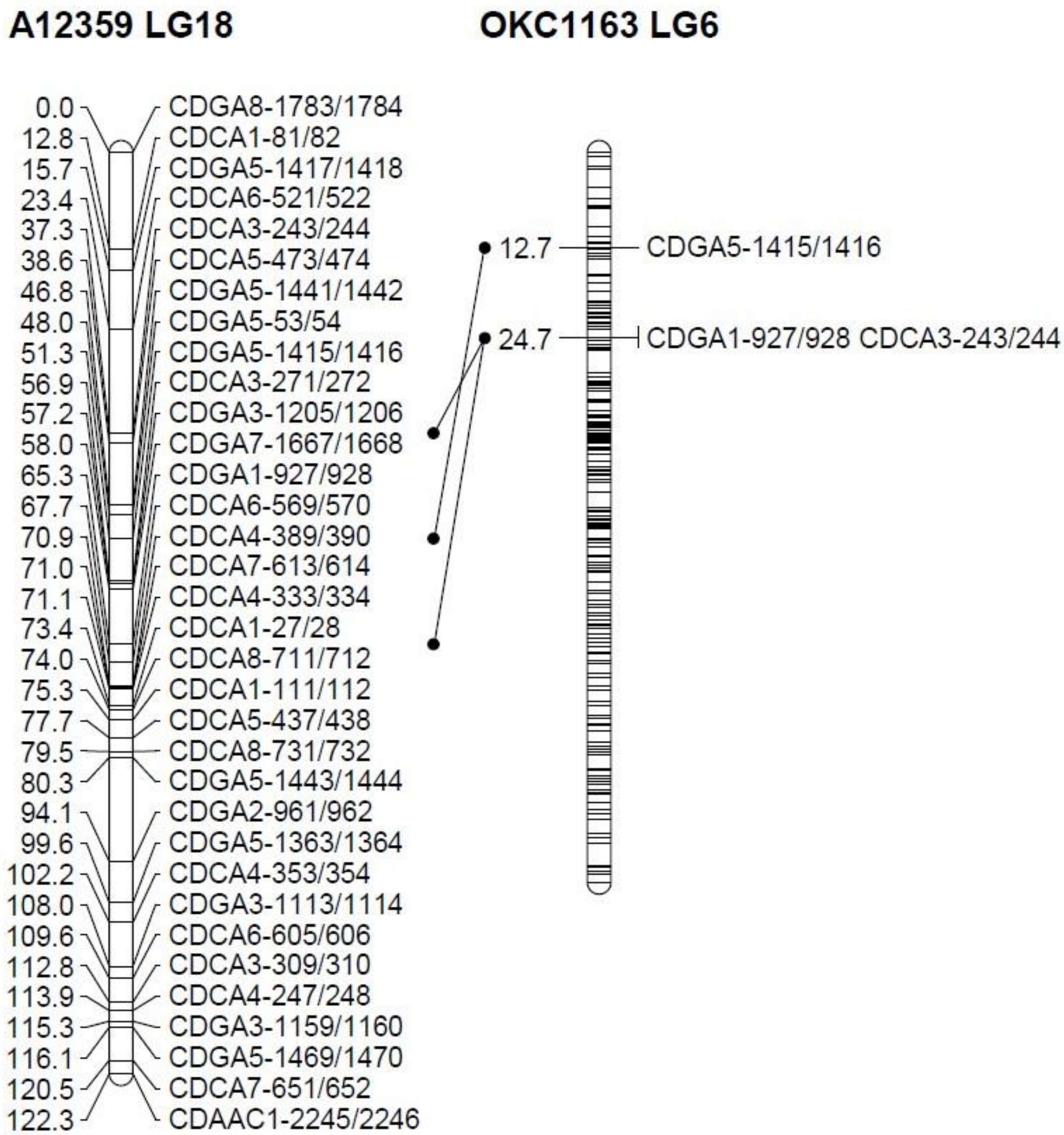

Fig. S5. *Cynodon transvaalensis* LG6 corresponding to *Cynodon dactylon* LG18 (Guo *et al*., 2017).

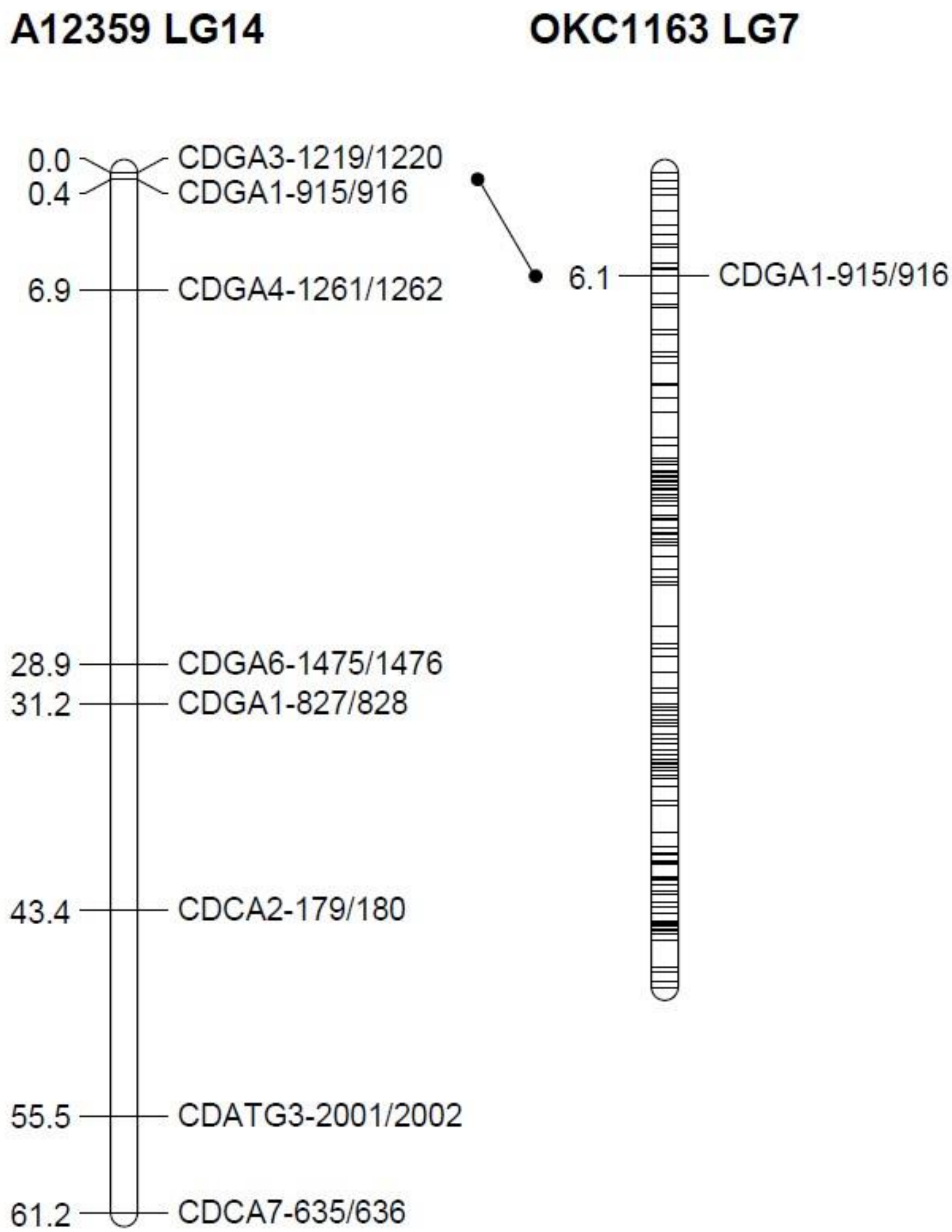

Fig. S6. *Cynodon transvaalensis* LG7 corresponding to *Cynodon dactylon* LG14 (Guo *et al*., 2017).

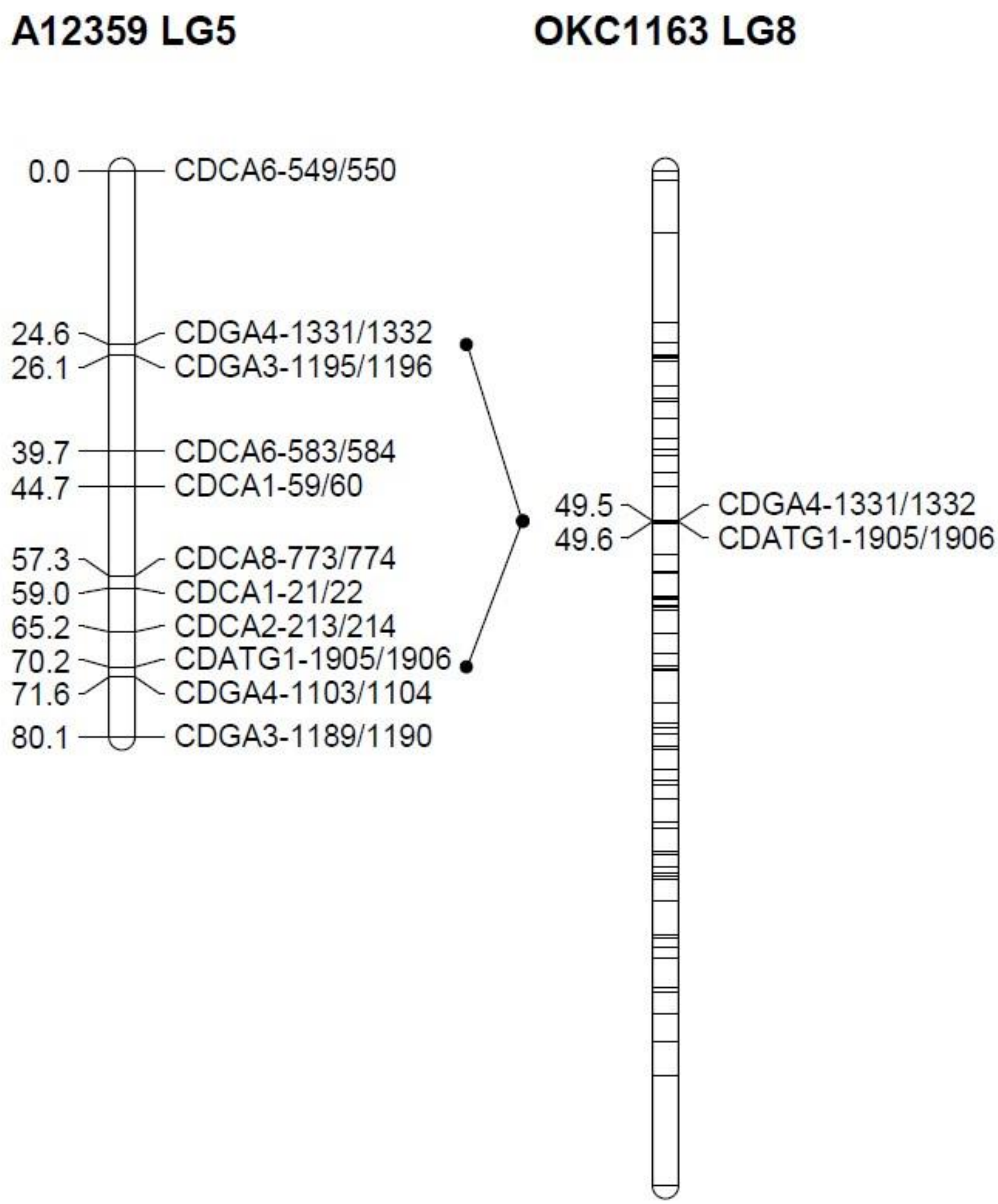

Fig. S7. *Cynodon transvaalensis* LG8 corresponding to *Cynodon dactylon* LG5 (Guo *et al*., 2017).

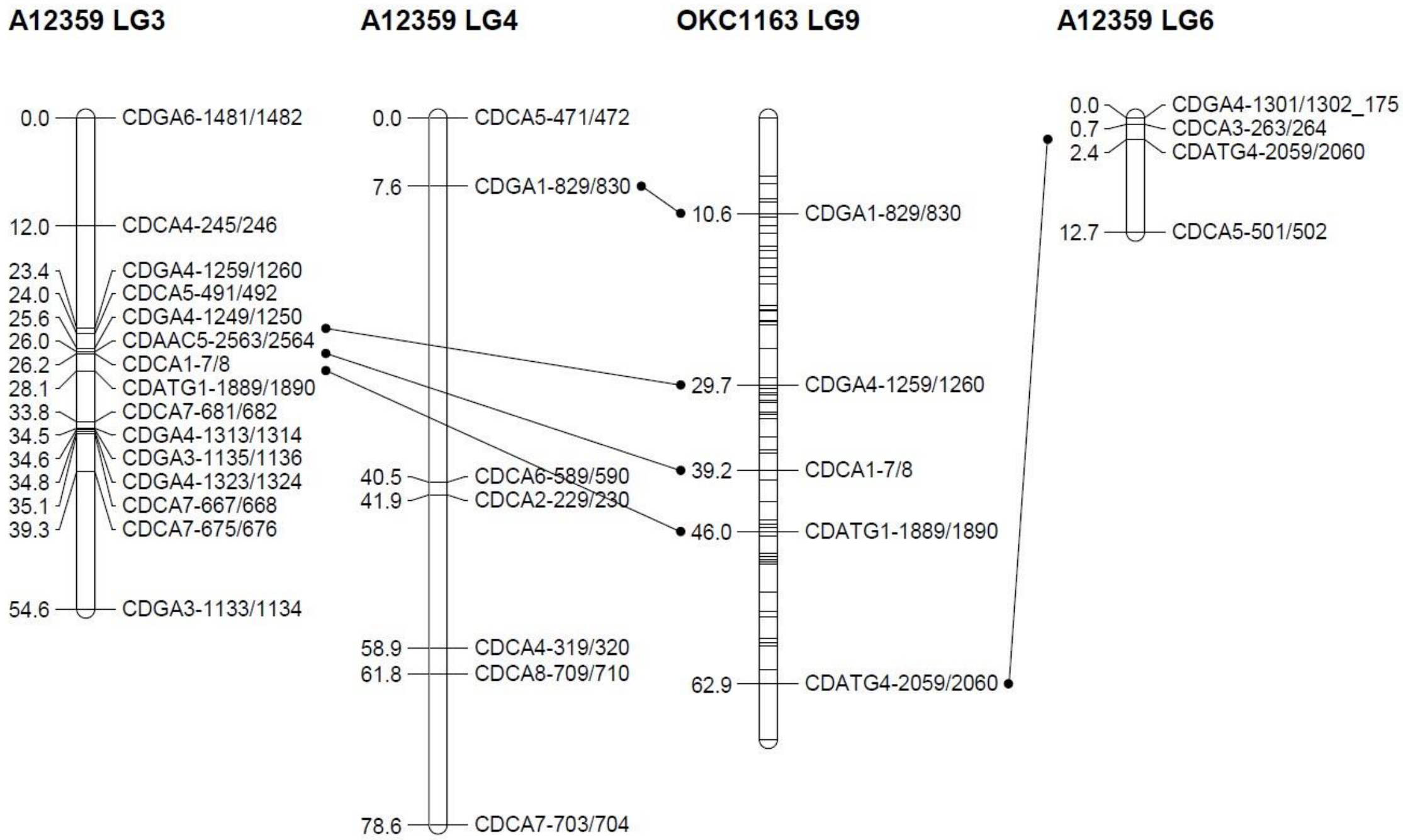

Fig. S8. *Cynodon transvaalensis* LG9 corresponding to *Cynodon dactylon* LGs3, 4, and 6 (Guo *et al*., 2017).
